# Velcrin molecular glues induce apoptosis in glioblastomas with high *PDE3A* and *SLFN12* expression

**DOI:** 10.1093/noajnl/vdae115

**Published:** 2024-07-01

**Authors:** Elisa Aquilanti, Silvia Goldoni, Andrew Baker, Kristyna Kotynkova, Sawyer Andersen, Vincent Bozinov, Galen F Gao, Andrew D Cherniack, Martin Lange, Ralf Lesche, Charlotte Kopitz, Philip Lienau, Timothy A Lewis, Marine Garrido, Stefan Gradl, Henrik Seidel, Yuen-Yi Tseng, Keith L Ligon, Patrick Y Wen, Matthew Meyerson, Heidi Greulich

**Affiliations:** Cancer Program, Broad Institute, Cambridge, Massachusetts, USA; Division of Neuro-Oncology, Department of Medical Oncology, Dana Farber Cancer Institute, Boston, Massachusetts, USA; Department of Medical Oncology, Dana Farber Cancer Institute, Boston, Massachusetts, USA; Bayer Pharmaceuticals, Research and Early Development Oncology, Cambridge, Massachusetts, USA; Department of Pediatric Oncology, Johns Hopkins School of Medicine, Baltimore, Maryland, USA; Cancer Program, Broad Institute, Cambridge, Massachusetts, USA; Cancer Program, Broad Institute, Cambridge, Massachusetts, USA; Cancer Program, Broad Institute, Cambridge, Massachusetts, USA; School of Medicine, University of Texas Southwestern, Dallas, Texas, USA; Cancer Program, Broad Institute, Cambridge, Massachusetts, USA; Department of Medical Oncology, Dana Farber Cancer Institute, Boston, Massachusetts, USA; Nuvisan ICB GmbH, Therapeutic Research, Berlin, Germany; Research and Development, Pharmaceuticals, Bayer AG, Berlin, Germany; Nuvisan ICB GmbH, Therapeutic Research, Berlin, Germany; Research and Development, Pharmaceuticals, Bayer AG, Berlin, Germany; Nuvisan ICB GmbH, Therapeutic Research, Berlin, Germany; Research and Development, Pharmaceuticals, Bayer AG, Berlin, Germany; Research and Development, Pharmaceuticals, Bayer AG, Berlin, Germany; Center for the Development of Therapeutics, Broad Institute, Cambridge, Massachusetts, USA; Bayer Consumer Care AG, Research and Early Development Oncology, Basel, Switzerland; Research and Development, Pharmaceuticals, Bayer AG, Berlin, Germany; Research and Development, Pharmaceuticals, Bayer AG, Berlin, Germany; Cancer Program, Broad Institute, Cambridge, Massachusetts, USA; Department of Pathology, Brigham and Women’s Hospital, Boston Children’s Hospital, Dana Farber Cancer Institute, Boston, Massachusetts, USA; Division of Neuro-Oncology, Department of Medical Oncology, Dana Farber Cancer Institute, Boston, Massachusetts, USA; Department of Genetics, Harvard Medical School, Boston, Massachusetts, USA; Cancer Program, Broad Institute, Cambridge, Massachusetts, USA; Department of Medical Oncology, Dana Farber Cancer Institute, Boston, Massachusetts, USA; Cancer Program, Broad Institute, Cambridge, Massachusetts, USA; Department of Medical Oncology, Dana Farber Cancer Institute, Boston, Massachusetts, USA

**Keywords:** glioblastoma, molecular glue, PDE3A, SLFN12, targeted therapy, Velcrin

## Abstract

**Background:**

Velcrins are molecular glues that kill cells by inducing the formation of a protein complex between the RNase SLFN12 and the phosphodiesterase PDE3A. Formation of the complex activates SLFN12, which cleaves tRNA^Leu^(TAA) and induces apoptosis. Velcrins such as the clinical investigational compound, BAY 2666605, were found to have activity across multiple solid tumor cell lines from the cancer cell line encyclopedia, including glioblastoma cell lines. We therefore aim to characterize velcrins as novel therapeutic agents in glioblastoma.

**Materials and Methods:**

*PDE3A* and *SLFN12* expression levels were measured in glioblastoma cell lines, the Cancer Genome Atlas (TCGA) tumor samples, and tumor neurospheres. Velcrin-treated cells were assayed for viability, induction of apoptosis, cell cycle phases, and global changes in translation. Transcriptional profiling of the cells was obtained. Xenograft-harboring mice treated with velcrins were also monitored for survival.

**Results:**

We identified several velcrin-sensitive glioblastoma cell lines and 4 velcrin-sensitive glioblastoma patient-derived models. We determined that BAY 2666605 crosses the blood-brain barrier and elicits full tumor regression in an orthotopic xenograft model of GB1 cells. We also determined that the velcrins BAY 2666605 and BRD3800 induce tumor regression in subcutaneous glioblastoma PDX models.

**Conclusions:**

Velcrins have antitumor activity in preclinical models of glioblastoma, warranting further investigation as potential therapeutic agents.

**Key Points**

- Velcrins are a novel class of molecular glues with anticancer activity in glioblastoma cells with high *PDE3A* and *SLFN12* expression.
- Velcrin treatment leads to tumor regression in vivo in orthotopic and subcutaneous preclinical glioblastoma models.

**Importance of the Study**

There is a significant unmet need for the development of novel targeted therapeutics to treat glioblastoma. Here, we demonstrate that velcrins are selectively toxic to glioblastoma cell lines and tumor neurospheres with high *PDE3A* and *SLFN12* expression. We also demonstrate that the velcrin BAY 2666605 is a CNS-penetrant compound that leads to tumor regression and prolonged survival in an orthotopic glioblastoma model. Lastly, BAY 2666605 and BRD3800 lead to tumor regression in subcutaneous glioblastoma patient-derived xenografts. These results warrant further investigation of velcrins as therapeutic agents in glioblastoma.

Glioblastoma is a highly aggressive cancer that has seen minimal therapeutic progress over the last decade.^1,2^ Survival rates remain low despite available therapeutic interventions, consisting of surgery, radiation therapy, and chemotherapy.^3,4^ Therefore, there is a significant unmet need for the development of targeted therapeutics for glioblastoma. Targeted cancer therapies traditionally consist of agents directed at somatic genomic alterations, such as tyrosine kinase inhibitors for non-small cell lung cancers with EGFR mutations or oncogenic ALK fusions. Although glioblastoma tumors are known to have oncogenic mutations in receptor tyrosine kinase genes,^5,6^ most targeted therapies have not shown clinical efficacy in this cancer, with the exception of the dabrafenib and trametinib combination in patients with tumors harboring BRAF V600E mutations, which was approved by the Food and Drug Administration in 2022.^7^

Over the last decade, novel mechanisms of cancer cell killing by small molecules have come to light, including “molecular glues,” or bifunctional small molecules that bring proteins into proximity and alter their native function.^8–10^ Recently, a differential viability screening approach coupled with chemogenomic analysis revealed a novel class of molecular glues, the “velcrins,” which kill cancer cells with high expression of the cyclic nucleotide phosphodiesterase, PDE3A, and of schlafen family member 12 (SLFN12).^11^ Velcrins induce the formation of a complex between PDE3A and SLFN12.^12,13^ This interaction is thought to increase SLFN12 protein stability in the cytoplasm,^14^ as well as to induce dephosphorylation of SLFN12.^15^ As a result, specific RNase activity of SLFN12 towards tRNA^Leu^(TAA) is induced. This in turn leads to ribosome pausing at TTA codons, which halts protein synthesis and induces apoptosis (Figure 1A).^16,^^^17^^

**Figure 1.**
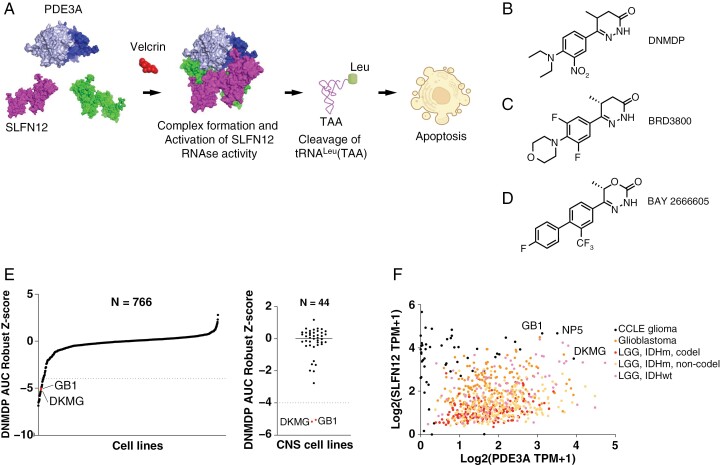
Biomarker expression and velcrin sensitivity in glioblastoma cell lines**. (**A) Schematic of the mechanism of cancer cell death induced by velcrins. Images were adapted from Ref. 17. (B) Chemical structure of DNMDP. (C) Chemical structure of BRD3800. (D) Chemical structure of BAY 2666605. (E) AUC robust Z-score values of sensitivity to DNMDP across a panel of cancer cell lines from Cancer Cell Line Encyclopedia (CCLE). Cell lines with Z-scores less than −4 were previously identified as sensitive.^11^ 2 out of 44 glioblastoma cell lines are sensitive to DNMDP. (F) *SLFN12* and *PDE3A* expression in glioblastoma cell lines from CCLE and glioma tumors from The Cancer Genome Atlas.

The first velcrin to be discovered to have PDE3A/SLFN12 complex-inducing activity was 6-(4-(diethylamino)-3-nitrophenyl)-5-methyl-4,5-dihydropyridazin-3(2H)one, or DNMDP^11^ (Figure 1B), and velcrin compounds have since been generated with improved pharmacokinetic properties,^18^ including BRD3800 (Figure 1C). One of these compounds, BAY 2666605 (Figure 1D), is a potent PDE3A/SLFN12 complex inducer and cytotoxic compound with IC_50_s in the low nanomolar range, suitable for testing in clinical trials.^19^ Here, we establish that BAY 2666605 is a central nervous system-penetrant compound. Additionally, we demonstrate the preclinical efficacy of BAY 2666605 and BRD3800 in glioblastomas with high expression of *PDE3A* and *SLFN12* in vitro and in vivo, in both cellular and patient-derived glioblastoma models.

## Materials and Methods

### Ethics Statement

Intracranial xenograft animal studies were performed in compliance with the guidelines and regulations of the Broad Institute Institutional Animal Care and Use Committee. Subcutaneous PDX animal studies were performed at EPO GmbH in Berlin, Germany, in compliance with the European Animal Welfare Act and German regional guidelines and regulations.

### Public Datasets

Results of the DNMDP sensitivity screen were downloaded from a previously published study.^11^ mRNA expression data for *SLFN12* and *PDE3A* in cells from CCLE were downloaded from the DepMap portal (https://depmap.org/portal/). TCGA mRNA expression data was downloaded from the GDC portal (https://portal.gdc.cancer.gov/).

### Cell Lines

Glioma cell lines (H4, DBTRG-05-MG, M059J, SW1783, GB1, DKMG, GAMG, LN229, NP5, T98G, U118 MG, U251 MG, and YKG1) were purchased from commercial sources. They were maintained in cell culture media supplemented with 10% fetal bovine serum and penicillin-streptomycin. Brain tumor neurospheres were obtained from the Dana Farber Center for Patient-Derived Models and the Broad Institute Cancer Cell Line Factory. They were cultured in Neural Stem Cell media supplemented with epidermal growth factor and fibroblast growth factor at 20 ng/mL as well as 0.2% heparin. BT856 was cultured in DMEM with 7.5% fetal bovine serum and 10 ng/mL EGF, 5 μg/mL insulin, 0.4 μg/mL hydrocortisone, 10 μM Y-27632, penicillin-streptomycin, and fungizone. Human bone marrow CD34 + cells were purchased from StemCell (70002.1) and cultured in StemSpan SFEM II media (09605) with 10 ng/mL TPO, SCF, FLT3L, IL3 and IL6. Human astrocytes were purchased from ScienCell (1800) and cultured in an astrocyte medium (1801). Human brain microvascular endothelial cells were purchased from ScienCell (1000) and cultured in endothelial cell medium (1001). All mycoplasma tests were negative at the time the experiments were conducted.

### Compounds

DNMDP was obtained from the Broad Institute Center for the Development of Therapeutics. BRD3800 and BAY 2666605 were obtained from Bayer AG. MG132 was purchased from Sigma Aldrich (M8699). Trequinsin was obtained from Sigma Aldrich (382425).

### Cell Viability Studies

Glioblastoma cell lines were seeded at a density of 600–1000 cells/well in 384-well plates. The following day, they were treated with the selected compounds and viability assessment was made after 72 hours using the CellTiterGlo reagent (Promega G7571). Glioblastoma neurospheres were seeded either in neurosphere form (BT856 and BT228) or on laminin-coated plates (BT288L, BT294, and BT359) at a density of 1000 cells/well in 96-well plates. A viability assessment was performed after 5 days using the CellTiterGlo reagent. Human astrocytes, endothelial cells, and bone marrow stem cells were seeded at a density of 2000 cells/well in 96-well plates and viability assessment was performed after 72 hours using the CellTiterGlo reagent.

### Immunoblots

Protein lysates were prepared using CHAPS lysis buffer with protease inhibitor (Millipore Sigma 11697498001) and 2.5 mM MgCl_2_. Samples were transferred to a PVDF membrane (Millipore Sigma IPVH00010). The following antibodies were used: anti-PDE3A (Bethyl A302-740A), anti-SLFN12 (ab234418), anti-actin (CST3700), and anti-GAPDH (sc-365062).

### Real-Time PCR

Total RNA was extracted from tumors and 1 μg of RNA was used for the reverse transcriptase reaction. Real-time PCR products were detected using SYBR green dye and primers targeting *SLFN12* (forward primer TTGGAAACGAATTATGCCGAGT, reverse primer AGAGCACACATAGCTCGTGAG), *PDE3A* (forward primer CCACGGCCTCATTACCGAC, reverse primer TTGCTCACGGCTCTCAAGG) as well as actin (*ACTB*; forward primer CATGTACGTTGCTATCCAGGC, reverse primer CTCCTTAATGTCACGCACGAT) and glyceraldehyde 3-phosphate dehydrogenase (*GAPDH*; forward primer ATGGGGAAGGTGAAGGTCG, reverse primer GGGGTCATTGATGGCAACAAT) as controls.

### Cell Cycle Assay

Cell cycle assays were performed as previously described.^20^ Cells were seeded at a density of 250 000 cells/well in 6-well plates and treated with DMSO or 100 nM BAY 2666605 for 36 hours. They were then fixed with 70% ethanol and resuspended in a staining solution of 100 μg/mL RNase A and 50 μg/mL propidium iodide in PBS. Data was collected using a Beckman CytoFLEX flow cytometer (2000 events per sample) and analyzed using FloJo.

### Apoptosis Assay

An apoptosis assay was performed by staining cells with FITC-bound Annexin V and propidium iodide (Thermo Fisher V13242). Cells were seeded at a density of 250 000 cells/well in 6-well plates and treated with DMSO or 100 nM BAY 2666605. 48 hours later, cells were harvested, resuspended in Annexin binding buffer, and stained with FITC-bound Annexin V and propidium iodide. Data was collected using a Beckman CytoFLEX (2000 events per sample) and analyzed using FlowJo.

### Measurement of Nascent Transcripts

This assay was performed as previously described.^16^ Cells treated with DMSO, 1 μM DNMDP or 1 μM BAY 2666605 for 2 hours were washed twice with PBS and incubated with 25 μM AHA in methionine-free RPMI for 4 hours. Cells were then lysed, and lysates were incubated with biotin alkyne, copper sulfate, and reducing agent from Click and Go Protein Capture Kit (Click Chemistry Tools 1440) for 90 minutes. Lysates were then run on SDS-PAGE gels, transferred to a PVDF membrane, and visualized by Ponceau S staining solution as a loading control. Biotinylated peptides were visualized using fluorescently labeled streptavidin (LICOR, 926-32230). Total protein and nascent peptides were quantified using ImageJ software.

### RNA Sequencing

Glioma cells were treated with 100 nM BAY 2666605 or DMSO for 24 hours. After RNA extraction, library synthesis was performed using the TruSeq mRNA HT Synthesis Kit (Illumina, RS-122-2103). Sequencing was performed with the Illumina HiSeq2500 machine. The number of replicates run for each condition are shown in Supplementary Figure 5A. The R DeSeq2 package generated gene log fold changes and adjusted *P* values for each cell line by comparing BAY 2666605-treated and control DMSO samples using RSEM expected counts. In each cell line, only genes with base mean over 10 were used for differential expression analysis.

### Central Nervous System Penetration Studies

Total plasma and brain tissue levels of BAY 2666605 were measured after a single oral dose of 10 mg/kg in immunocompromised nude mice (Nu/Nu). Measurements were taken using liquid chromatography-tandem mass spectrometry (LC-MS-MS) at 0.5, 1, 3, 6, 24, and 48 hours after compound administration. Total levels were corrected for binding in mouse plasma and brain tissue homogenate and plotted over time.

### Binding to Plasma Proteins and Tissue homogenate

HT equilibrium dialysis was used to determine the binding in mouse plasma and brain homogenate as previously outlined.^21^ In brief, a semipermeable membrane separated the plasma/tissue homogenate from the buffer compartment. The test compound was added to the plasma side at a concentration of 3 μM and incubated for 7 hours at 37°C and 5% CO2 with 99% humidity and moderate shaking. Then 10 μL of the plasma side was transferred to a deep-well plate containing 90 μL of buffer and 90 μL of the buffer side was added to 10 μL of blank plasma. All samples were precipitated with 400 μL ice-cold MeOH and frozen overnight at −20°C. After thawing and mixing, the samples were centrifuged for 10 minutes at 3000 rpm. The supernatant was transferred to a 96-well plate, and LC-MS/MS measurements were undertaken. From the quotient of buffer and plasma concentration, the unbound fraction was calculated.

### Intracranial Mouse Injections

Intracranial mouse injections were performed as previously described.^22^ Briefly, 6-week-old female SCID mice (CB17/Icr-*Prkdc*^*scid*^/IcrIcoCrl) were obtained from Charles River Laboratories. Animals were first anesthetized with isoflurane; the surgical field was then prepared by trimming hair and disinfecting with ethanol and betadine. A 1 cm incision was made in the scalp and the skull was penetrated using a drill with a 1.4 mm burr, 2 mm to the right of the bregma. Coordinates were obtained using a stereotactic device. 300 000 cells in 2 μL of PBS were injected at a 2 mm depth. The surgical site was then closed by suturing. Perioperative care was administered using a subcutaneous injection of 1 mg/kg buprenorphine after the procedure and 3 daily injections of 1 mg/kg meloxicam starting the day of surgery. Animals were euthanized once they reached humane endpoints.

### Compound Administration in Mice

BAY 2666605 was diluted in 5% ethanol in polyethylene glycol (PEG). It was administered using oral gavage at doses of 10 mg/kg twice a day. Control animals received 5% ethanol in PEG via oral gavage twice daily.

### Intracranial Tumor Imaging

Animals were first anesthetized using isoflurane; they then received intra-peritoneal injections of luciferin at 150 mg/kg. Bioluminescence images were captured using the Perkin Elmer in vivo imaging system. Luminescence was quantified using the Living Image Software.

### Subcutaneous Glioblastoma Models

Glio11305 and Glio12421 were obtained from EPO GmbH. Transcriptomic analysis of glioblastoma PDX models was performed at EPO GmbH. 10 6-week-old NOD.Gg-*Prkdc*^scid^*Il2rg*^tm1Sug^/JicTac (NOG) mice were injected subcutaneously with Glio11305 and Glio12421 cells. Flank tumor size was then measured weekly, and animals were randomized once tumor size reached 0.1–0.2 cm^3^. At the time of randomization, 5 animals received 10% ethanol in polyethylene glycol (PEG400) twice daily through oral gavage, and 5 animals received 5 mg/kg of BAY 2666605 twice daily via oral gavage, or 40 mg/kg BRD3800 twice daily. Treatment was continued until control animals were sacrificed. Animals were sacrificed once tumor volume reached 1 cm^3^.

### Statistical Analysis

**Figure 2.**
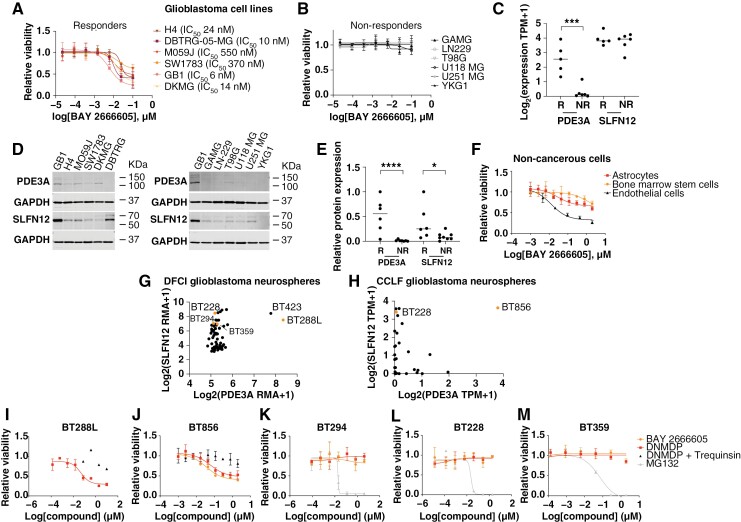
Velcrin treatment reduces viability in glioblastoma models with high *PDE3A* and *SLFN12* expression. (A) BAY 2666605 dose–response curves and IC_50_ values of glioblastoma cell lines that are velcrin-sensitive. (B) Dose–response curves of glioblastoma cell lines that are velcrin-insensitive. (C) *PDE3A* and *SLFN12* mRNA expression of glioblastoma cell lines that are velcrin-sensitive versus velcrin-insensitive. (D) Immunoblot showing PDE3A and SLFN12 protein expression in glioblastoma cell lines that are velcrin-sensitive and vecrin-insensitive. (E) Quantification of PDE3A and SLFN12 protein expression (normalized to GB1 cells) in glioblastoma cell lines that are velcrin-sensitive and velcrin-insensitive. (F) BAY 2666605 dose–response curves in normal human astrocytes, human bone marrow stem cells, and brain microvascular endothelial cells. (G) *PDE3A* and *SLFN12* mRNA expression were obtained using microarrays in a panel of glioblastoma neurospheres from DFCI CPDM. (H) *PDE3A* and *SLFN12* mRNA expression was obtained using RNA sequencing in a panel of glioblastoma neurospheres from Broad Institute CCLF. (I). Dose–response curve of BT288L treated with DNMDP and DNMDP with 100 nM trequinsin. (J) Dose–response curve of BT856 treated with DNMDP, BAY2666605, and DNMDP with 100 nM trequinsin. (K) Dose–response curve of BT294 treated with DNMDP, BAY 2666605, and MG132. (L) Dose–response curve of BT228 treated with DNMDP, BAY 2666605, and MG132. (M) Dose–response curve of BT359 treated with DNMDP, BAY 2666605, and MG132. *=*P* < .05, ***=*P* < .001, ****=*P* < .0001.

**Figure 3.**
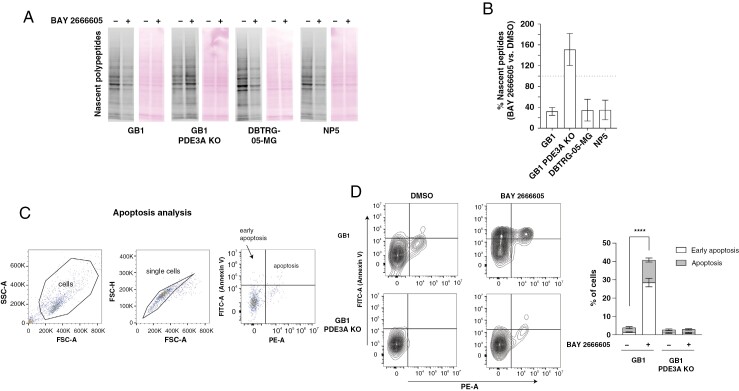
Velcrin treatment halts translation and induces apoptosis in sensitive glioblastoma cell lines. (A) Immunoblot showing the relative amounts of nascent polypeptides (left) and Ponceau-stained controls (right) in GB1, GB1 PDE3A KO, DBTRG-05-MG, and NP5 cells treated with 100 nM BAY 2666605 versus DMSO. (B) Quantification of nascent polypeptides in GB1, GB1 PDE3A KO, DBTRG-05-MG, and NP5 cells treated with BAY 2666605 relative to DMSO. (C) Schematic of flow cytometry-based apoptosis assay analysis, performed by staining cells with FITC-bound Annexin V and Propidium Iodide. Cells that only stain positive for Annexin V are undergoing early apoptosis, whereas cells staining positive for both Annexin V and Propidium Iodide are apoptotic. (D) Results of apoptosis assay in GB1 and GB1 PDE3AKO cells treated with DMSO and 100 nM BAY 2666605. ****=*P* < .0001.

**Figure 4.**
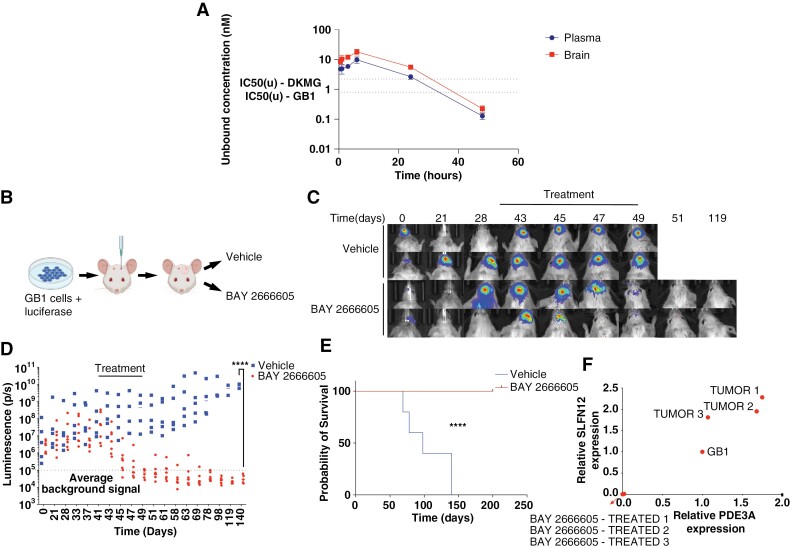
BAY 2666605 is a CNS-penetrant compound that leads to glioblastoma tumor regression in a murine model. (A) Brain and plasma concentrations of unbound BAY 2666605 after a single oral dose of 10 mg/kg BAY 2666605. Measurements were taken 0.5, 1, 3, 6, 24, and 38 hours after dosing. (B) Schema of the experimental approach to test BAY 2666605 anticancer activity in vivo in an orthotopic glioblastoma murine model. (C) Representative serial images showing intracranial luminescence of animals orthotopically engrafted with GB1 cells and treated with vehicle or BAY 2666605. (D) Quantification of intracranial luminescence of animals treated with vehicle or BAY 26666605. (E) Survival curves of animals treated with vehicle or BAY2666605. (F) Relative expression levels of *PDE3A* and *SLFN12* in GB1 cells grown in culture, intracranial tumors derived from GB1 cells in animals treated with vehicle, and right brain hemisphere of animals harboring intracranial tumors treated with BAY 2666605. **** = *P* < .0001.

**Figure 5.**
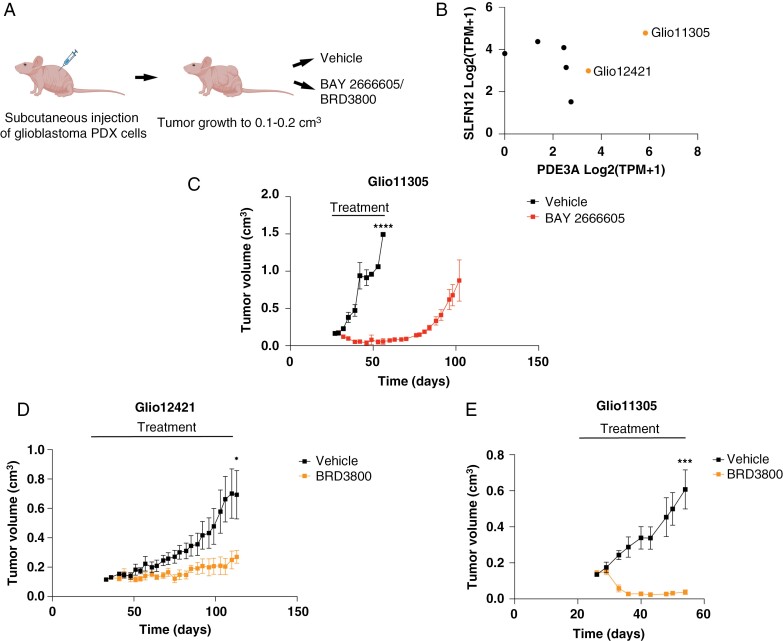
Velcrin treatment leads to tumor regression in vivo in glioblastoma PDX models. (A) Schema of the experimental approach to test velcrin activity in vivo in glioblastoma subcutaneous PDX models. (B) *PDE3A* and *SLFN12* mRNA expression of glioblastoma PDX models. (C) Serial quantification of tumor volumes in animals harboring subcutaneous Glio11305 tumors treated with vehicle or twice daily doses of 5 mg/kg BAY 2666605. (D) Serial quantification of tumor volumes in animals harboring Glio12421 tumors treated with vehicle or twice daily doses of 40 mg/kg BRD3800. (E) Serial quantification of tumor volumes in animals harboring Glio11305 tumors treated with vehicle or twice daily doses of 40 mg/kg BRD3800. * = *P* < .05, *** = *P* < .001, **** = *P* < .0001.

Data in all the graphs shown represent the average of biological or technical replicates as indicated in the figure legends, and error bars represent standard deviations. For Figures 2C and E, 3D, 4D, 5C, D, and E, *P*-values were calculated using the unpaired *t*-test (GraphPad Prism 9). For Figure 4E, survival analysis was performed using the Kaplan–Meier method and *P*-values were calculated using the Log-Rank test (GraphPad Prism 9).

## Results

### Characterization of Velcrin Sensitivity and Biomarker Expression in Glioblastoma Models

To determine cancer types that are sensitive to velcrin treatment, we analyzed previously published data from a DNMDP sensitivity screen across 766 cancer cell lines from the Cancer Cell Line Encyclopedia (CCLE).^11^ This screen unveiled 22 DNMDP-sensitive lines with robust Z-scores < −4.^11^ Among these lines were 2 glioblastoma cell lines, GB1 and DKMG (Figure 1E, left panel). In total, 2 out of 44 glioblastoma cell lines included in the screen (~5%) were sensitive to DNMDP (Figure 1E, right panel). GB1, DKMG, and NP5 were among the CCLE glioblastoma cell lines with the highest *PDE3A* and *SLFN12* expression, consistent with prior reports (Figure 1F).^11^ Next, we compared *PDE3A* and *SLFN12* expression in the CCLE cell lines with expression values from primary gliomas from the Cancer Genome Atlas (TCGA; Figure 1F, Supplementary Table 1). We found that a subset of tumors harbors high expression of *PDE3A* and *SLFN12*, with comparable levels to the CCLE cell lines. Tumors with the highest *PDE3A* and *SLFN12* expression levels include *IDH* wild-type low-grade gliomas and glioblastomas.

To validate the response to velcrin treatment in biomarker-positive glioblastoma cell lines and to determine whether the response is dependent on PDE3A, we tested velcrin sensitivity in GB1 cells and GB1 cells where PDE3A was ablated using CRISPR-Cas9 (GB1 PDE3A KO; Supplementary Figure 1A). GB1 cells were sensitive to both DNMDP and BAY 2666605 (Supplementary Figure 1B), consistent with prior reports,^11,12^ and loss of viability was rescued by co-treatment with 100 nM of trequinsin, a potent PDE3A inhibitor that does not induce cellular toxicity and competes with velcrins for the active site of PDE3A^11,12^. GB1 PDE3A KO cells were insensitive to either compound, but were killed by the proteasome inhibitor MG132, which is a known broadly cytotoxic agent (Supplementary Figure 1C). Next, we tested a panel of commercially available glioma cell lines with variable levels of *PDE3A* expression for sensitivity to BAY 2666605. We identified 6 responding cell lines in addition to GB1 (H4, DBTRG-05-MG, M059J, SW1783, DKMG, and NP5) with IC_50_s ranging from 6 to 550 nM (Figure 2A, B, Supplementary Figure 2), and detected a strong correlation between *PDE3A* expression and response to BAY 2666605 (Figure 2C). Velcrin-sensitive-cell lines also exhibited higher protein expression levels of PDE3A and SLFN12 compared to velcrin-insensitive cell lines (Figure 2D), and a correlation was detected between both PDE3A and SLFN12 protein expression and sensitivity to velcrins (Figure 2E). Lastly, to characterize potential toxic effects of velcrin compounds in normal human cells, we treated astrocytes, bone marrow hematopoietic (CD34+) stem cells and human brain microvascular endothelial cells with BAY 2666605 (Figure 2F). Astrocytes exhibited a very mild (<20%) reduction in viability at concentrations in the nM range, bone marrow stem cells exhibited a mild reduction in viability at μM concentrations and the endothelial cells were sensitive to BAY 2666605 (Figure 2F).

In order to validate velcrin sensitivity in preclinical glioblastoma models that faithfully recapitulate genomic and transcriptomic profiles of primary tumors, we analyzed *PDE3A* and *SLFN12* expression levels in glioblastoma neurospheres^23^ from the Dana Farber Center for Patient-Derived Models and the Broad Institute Cancer Cell Line Factory (Figure 2G and H, Supplementary Table 2, 3 and Figure 3).^24^ We selected the neurosphere models, BT288L, BT856, BT294, BT228, and BT359 (Figure 2G and H, Supplementary Table 4), with a range of *PDE3A* expression levels, for validation of velcrin sensitivity. The neurosphere model BT423, which displays high *PDE3A* expression, did not grow in culture and therefore could not be included in our study. We found that BT288L and BT856 are sensitive to DNMDP with IC_50_s of 62 nM and 61 nM (Figure 2I and J), BT856 is sensitive to BAY2666605 with IC_50_ of 36 nM (Figure 2I, while BT294, BT228 and BT359, expressing lower levels of *PDE3A*, are not sensitive to either DNMDP or BAY2666605; Figure 2K, L, and M). It is worth noting that both BT856 and BT288L are partially sensitive models, with >10% of persister cells at the highest concentrations of the compound tested. To determine whether the loss of viability observed in BT288L and BT856 is dependent on PDE3A, we co-treated the cells with DNMDP and 100 nM trequinsin and found that trequinsin rescues the toxic effects of DNMDP (Figure 2I and J).

In summary, we demonstrated that velcrins have anti-proliferative effects in a subset of glioblastoma cell lines and neurosphere models and validated a correlation between *PDE3A* expression and response to velcrin treatment.

### Velcrins Induce Apoptosis and Halt Translation in Sensitive Glioblastoma Models

We next sought to elucidate the cellular mechanisms underlying the loss of viability observed in velcrin-sensitive glioblastomas. To determine whether the mechanism of growth inhibition in velcrin-sensitive glioblastoma cell lines is consistent with prior reports of global translation inhibition,^16^ we measured the relative abundance of nascent polypeptides in glioblastoma cell lines treated for 2 hours with DNMDP and 1 μM BAY 2666605, compared to DMSO. Velcrin treatment led to a significant reduction in nascent polypeptide formation in velcrin-sensitive glioblastoma cell lines GB1, DBTRG-05MG, and NP5, but not in GB1 PDE3A KO cells (Figure 3A and B). To determine whether velcrin treatment induces cell death and cell cycle arrest, we performed cell cycle and apoptosis assays in GB1 and GB1 PDE3A KO treated with DMSO and with 100 nM BAY 2666605. We detected a mild statistically significant reduction in G0/G1 and S phase and an increase in G2/M for GB1 cells treated with BAY 2666605, whereas no significant differences were detected for GB1 PDE3A KO cells (Supplementary Figure 4A, B). Additionally, we detected a substantial induction of apoptosis in GB1 cells treated with 100 nM BAY 2666605, whereas no apoptosis was detected for GB1 PDE3A KO cells (Figure 3C and D).

Lastly, we performed RNA sequencing and differential gene expression analysis in velcrin-sensitive (DBTRG-05-MG, GB1, DKMG) and insensitive (LN229, T98G, U118 MG) glioma cells treated with 100 nM BAY 2666605 or DMSO for 24 hours. While we detected thousands of differentially expressed genes in velcrin-sensitive cells treated with BAY 2666605 compared to DMSO, no genes to a few hundred genes were differentially expressed in velcrin-insensitive cells (Supplementary Figure 5A). Among 2814 genes significantly differentially expressed in all 3 sensitive-cell lines (*P* < .05), we detected an enrichment of 42 apoptosis pathway genes (Chi-Square *P* = .0028), supporting the previously established mechanism of cell death (Supplementary Figure 5B).

In summary, we determined that velcrin treatment induces cell death by apoptosis and halts translation in sensitive glioblastoma cells.

### In Vivo Efficacy of Velcrins in Preclinical Glioblastoma Models

To determine whether BAY 2666605 penetrates the blood-brain barrier, we measured unbound plasma and brain levels of BAY 2666605 after a single oral dose of 10 mg/kg in mice at serial time points (Figure 4A). We detected unbound BAY 2666605 concentrations up to 18 nM in both plasma and brain 6 hours after compound administration, after which levels began to decline (Figure 4A). These concentrations were comparable to the nM range IC_50_s we observed for in vitro drug sensitivity experiments with GB1 cells and glioblastoma neurospheres (Figure 2A and I).

We then sought to determine whether BAY 2666605 has antitumor activity in an orthotopic glioblastoma model. We performed orthotopic injections of GB1 cells harboring firefly luciferase into immunocompromised mice and serially measured intracranial luminescence until the average signal increased by one order of magnitude compared to baseline, indicating intracranial tumor formation. At that point, we initiated twice daily treatment with 10 mg/kg BAY 2666605 for 7 animals, while 5 animals received vehicle (Figure 4B). Treatment was continued for a total of 10 days. Shortly after treatment initiation, we detected a significant reduction in intracranial luminescence in animals that received BAY 2666605, while intracranial luminescence of animals in the control group continued to rise (Figure 4C, 4D). Strikingly, intracranial luminescence was fully ablated in all animals receiving BAY 2666605 and remained undetectable for up to 91 days after treatment discontinuation (Figure 4C, 4D). This in turn translated into a significant survival prolongation for animals treated with BAY 2666605, which remained alive for a total of 200 days from tumor implantation, at which point they were euthanized as per protocol. In contrast, all animals in the control group were dead 150 days after tumor implantation (Figure 4E).

To determine whether BAY 2666605-treated animals retained any *SLFN12*- and *PDE3A*-expressing GB1 cells in their brains at the time of euthanasia, we harvested intracranial tumors from 3 control animals and the right cerebral hemisphere from 3 animals treated with BAY 2666605, which is where tumor implantations were previously performed. We extracted total RNA from these tissues and performed real-time quantitative PCR for *SLFN12* and *PDE3A* using human-specific primers. While we detected human *SLFN12* and *PDE3A* expression in the intracranial tumors, we did not detect human *SLFN12* or *PDE3A* expression in the brains of animals treated with BAY 2666605, indicating that most *SLFN12*- and *PDE3A*-expressing cells were ablated by BAY 2666605 (Figure 4F).

Lastly, tested velcrin activity in glioblastoma patient-derived models in vivo. We selected glioblastoma models Glio11305 and Glio12421 and injected them subcutaneously in the flanks of immunocompromised mice (Figure 5A). These models are known to have high expression of *PDE3A* and *SLFN12* based on transcriptomic analysis (Supplementary Table 5, Figure 5B). Once tumors began to grow, we treated the animals with twice daily doses of BAY 2666605 at 5 mg/kg or BRD3800 at 40 mg/kg. We continued treatment until all animals in the control group were euthanized (once tumor volume reached ~1 cm^3^). We observed a near-total regression of the subcutaneous tumors in animals harboring Glio11305 tumors treated with BAY 2666605 (Figure 5B). Some of the tumors began to grow 3 weeks after treatment discontinuation (Figure 5C). Similarly, we observed tumor regression when animals harboring Glio11305 and Glio12421 tumors were treated with BRD3800 (Figure 5D, 5E). Administration of both BAY 2666605 and BRD3800 did not cause significant toxicity in the animals as evidenced by stable body weights and lack of symptoms of distress (Supplementary Figure 6).

Taken together, our results indicate that velcrins have antitumor activity in vivo in preclinical glioblastoma models.

## Discussion

Velcrins are a novel class of molecular glues that lead to the formation of a protein complex between PDE3A and SLFN12, activating RNase activity of SLFN12 and leading to tRNA^Leu^(TAA) cleavage and cessation of protein synthesis. Velcrins were found to have potent anticancer activity and selectively kill cancer cells with elevated *PDE3A* and *SLFN12* expression. The velcrin compound BAY 2666605 was recently evaluated in an early-phase clinical trial across multiple tumor types.

Here, we identified glioblastoma as a potential clinical indication space for velcrins. We characterized *PDE3A* and *SLFN12* expression in commercial glioblastoma cell lines and glioblastoma neurospheres.^23^ We validated velcrin sensitivity in a subset of both glioblastoma cell lines and glioblastoma neurospheres and determined that *PDE3A* expression is the key driver of sensitivity in both sets of models. We also confirmed that velcrin-treated glioblastoma cells display a reduction in nascent translation, consistent with prior reports, as well as apoptosis.^16^ Because of the limited number of models tested, the precise expression threshold for sensitivity in glioblastoma neurospheres is yet to be determined. To identify patients with velcrin-sensitive tumors for early-phase clinical trials, it will be necessary to develop specific assays to detect PDE3A and SLFN12 mRNA or protein expression. Lastly, it will be important to determine whether tumors with high *PDE3A* and *SLFN12* expression levels retain these features at recurrence, and whether *PDE3A* and *SLFN12* expression are affected by other therapeutic interventions.

To further evaluate the efficacy of BAY 2666605 in preclinical glioblastoma models in vivo, we tested its ability to access the central nervous system. We determined that this compound penetrates freely through the blood-brain barrier, with intracranial levels corresponding to plasma levels, and lasting up to 6 hours after compound administration. These results make BAY 2666605 a particularly promising agent, as the limited blood-brain barrier penetration of several chemotherapies and anticancer-targeted therapies has greatly hindered the development of novel therapeutics for glioblastoma.^2^ We also determined that BAY 2666605 has striking in vivo anticancer activity in an orthotopic xenograft model of GB1 cells, leading to full tumor regression and long-term survival of all treated mice. Lastly, we observed in vivo sensitivity to 2 velcrin compounds in subcutaneous glioblastoma PDX models with high *PDE3A* and *SLFN12* expression. While these results are encouraging, further study is required to investigate the in vivo efficacy of velcrins in orthotopic glioblastoma PDX models with a range of biomarker expressions, and to determine whether velcrins lead to full tumor regression and long-term survival in these models.

To conclude, it is worth discussing some potential limitations of velcrins as a cancer therapeutic strategy. First, it is likely that a relatively small percentage of glioblastoma patients will have velcrin-sensitive tumors. Based on the results presented in this work, 50% of tested cell lines were velcrin-sensitive, but only 3% of glioblastoma neurospheres were predicted to be sensitive (including BT423, which has similar *PDE3A* expression levels to BT288L). Additionally, analysis of TCGA tumor samples revealed that a small fraction of glioblastomas have biomarker expression comparable to velcrin-sensitive-cell lines. If the neurosphere data is reflective of patient data, approximately 400 patients will be diagnosed with velcrin-sensitive glioblastomas each year in the United States, based on the prediction of ~13 000 new glioblastoma diagnoses each year.^4^ Additional studies with glioblastoma patient-derived models will be required to determine the fraction of velcrin-sensitive tumors more accurately. A second limitation is that it is possible that tumors will become rapidly resistant to velcrin treatment. *SLFN12* and *PDE3A* are not known to play a role in oncogenesis or to be required for tumor maintenance, and they are not known to be essential genes for cell survival. It is therefore conceivable that resistance may develop by suppressing expression of *PDE3A* or *SLFN12*.^25^ This is especially concerning for glioblastoma, which is known to harbor a high degree of heterogeneity and plasticity.^26,27^ Lastly, the toxicity profile of BAY 2666605 in humans is yet to be reported. While animals treated with BAY 2666605 did not display obvious toxicities as evidenced by the absence of symptoms and stable body weights, it is challenging to extrapolate on-target toxicity of this compound from murine data as there is no known murine ortholog of SLFN12.^14^ It is possible, however, to predict on target, off mechanism toxicities related to PDE3A inhibition and off-target toxicities from murine data. It is worth noting that while we did not detect significant velcrin sensitivity in astrocytes and bone marrow stem cells, brain microvascular endothelial cells were found to be velcrin-sensitive, suggesting possible blood-brain barrier toxicity. If systemic dose-limiting toxicity is observed in human trials, this could be circumvented in patients with glioblastoma by using a local delivery strategy, including convection-enhanced delivery^28^ or intrathecal delivery.

Taken together, our work identifies velcrins as a novel therapeutic class with promising preclinical activity against glioblastomas with elevated *SLFN12* and *PDE3A* expression. Further study is required to determine mechanisms of resistance to velcrin compounds as well as possible toxicities.

## Supplementary Material

vdae115_suppl_Supplementary_Figures

vdae115_suppl_Supplementary_Tables

## Data Availability

*SLFN12* and *PDE3A* mRNA expression data for the CCLE glioblastoma cell lines and TCGA tumors is included in Supplementary Table 1. *SLFN12* and *PDE3A* mRNA expression data for the glioblastoma neurospheres is included in Supplementary Tables 2 and 3. *SLFN12* and *PDE3A* mRNA expression data for the subcutaneous PDX models is included in Supplementary Table 5. RNA sequencing data for glioma cell lines treated with BAY 2666605 versus DMSO has been deposited on the Sequence Read Archive.
