# Supplementary material for: Velcrin molecular glues induce apoptosis in glioblastomas with high PDE3A and SLFN12 expression
Source: Neurooncol Adv. 2024 Jul 1;6(1):vdae115. doi: 10.1093/noajnl/vdae115 (PMC11333922; doi:10.1093/noajnl/vdae115)
Supplement: vdae115_suppl_Supplementary_Figures [file vdae115_suppl_supplementary_figures.docx]

**Supplementary materials**


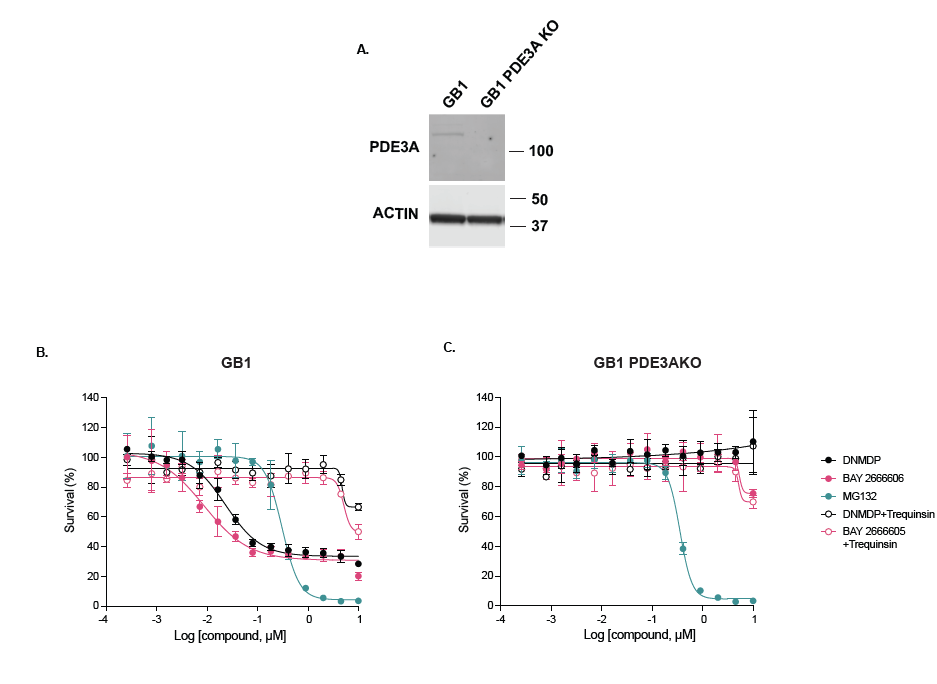


**Supplementary Figure 1:** Immunoblot showing PDE3A protein expression in GB1 cells and GB1 PDE3A KO cells. B. Dose response curves of GB1 cells treated with DNMDP, BAY 2666605, DNMDP or BAY 2666605 with 100 nM trequinsin, and MG132. C. Dose response curves of GB1 PDE3A KO cells treated with DNMDP, BAY 2666605, DNMDP or BAY 2666605 in combination with 100 nM Trequinsin and MG132.


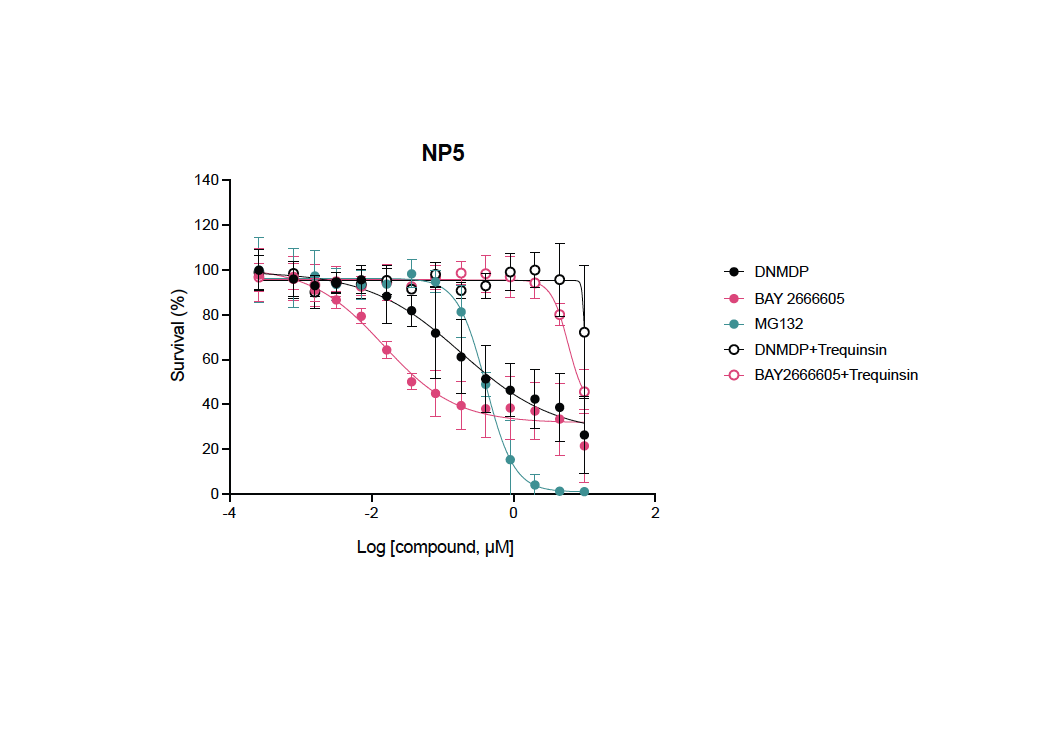


**Supplementary Figure 2:** Dose response curves of NP5 cells treated with DNMDP, BAY 2666605, DNMDP or BAY 2666605 in combination with 100 nM tA. Table summarizing replicate numbers for each condition for the RNA sequencing experiment. B. Table summarizing number of differentially expressed genes in glioblastoma velcrin-sensitive (DBTRG-05-MG, DKMG, GB1) and insensitive (LN229, T98G, U118 MG) cells treated with DMSO vs. 100 nM BAY 2666605, collected 24 hours post-treatment. B. 42 apoptosis pathway genes significantly up or down regulated (pAdj < 0.05 in all three velcrin sensitive-cell lines.


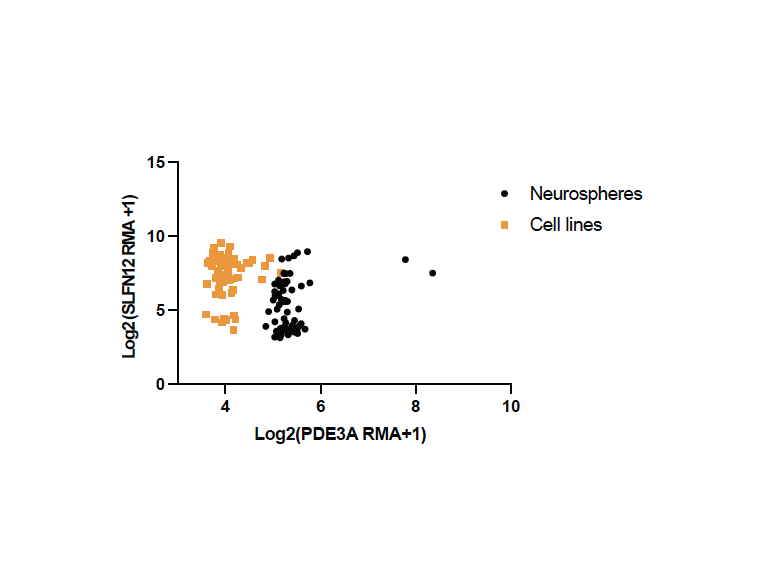


**Supplementary Figure 3:** Relative *PDE3A* and *SLFN12* mRNA expression in glioblastoma cell lines and glioblastoma neurospheres, measured using microarray technology.


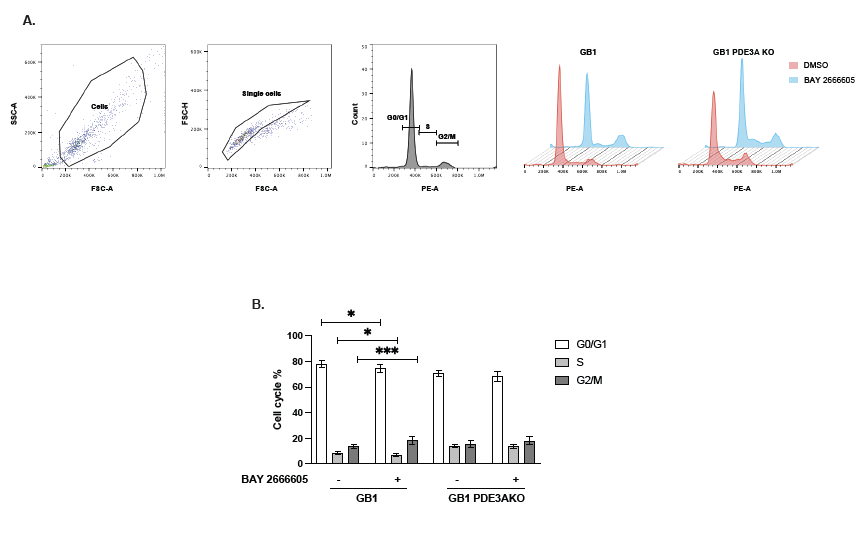


**Supplementary Figure 4:** A. Schematic of cell cycle analysis. B. Fraction of GB1 and GB1 PDE3A KO cells in G0/G1, S, and G2/M phases of the cell cycle treated with DMSO and 100 nM BAY 2666605 for 36 hours. * = p<0.05, *** = p<0.001

**
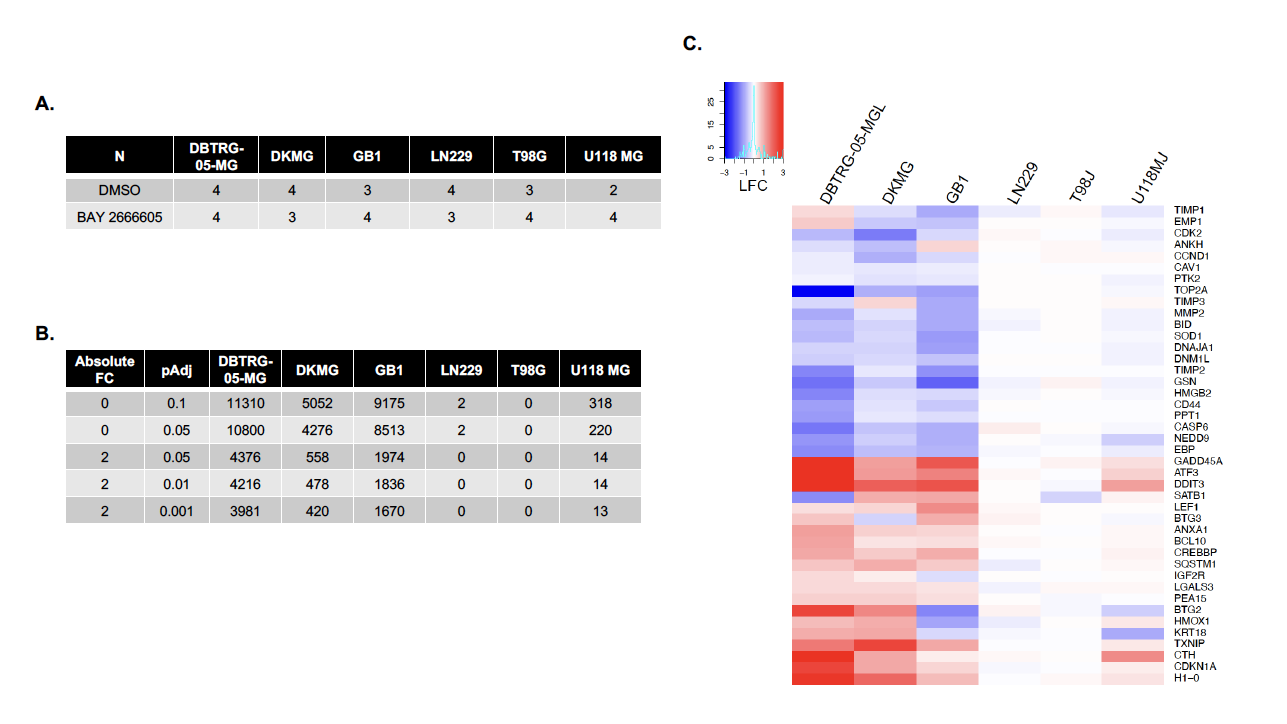
Supplementary Figure 5:** A. Table summarizing replicate numbers for each condition for the RNA sequencing experiment. B. Table summarizing number of differentially expressed genes in glioblastoma velcrin-sensitive (DBTRG-05-MG, DKMG, GB1) and insensitive (LN229, T98G, U118 MG) cells treated with DMSO vs. 100 nM BAY 2666605, collected 24 hours post-treatment. B. 42 apoptosis pathway genes significantly up or down regulated (pAdj < 0.05 in all three velcrin sensitive-cell lines.


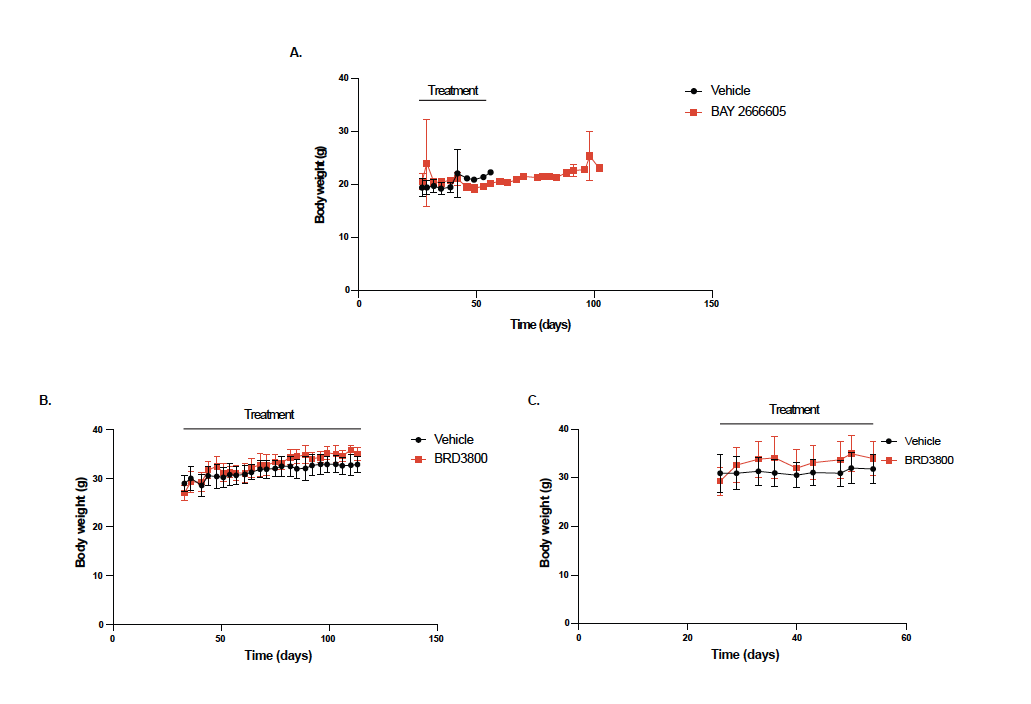


**Supplementary Figure 6:** A. Serial measurements of body weights in animals harboring Glio11305 tumors treated with vehicle and twice daily doses of BAY 2666605. B. Serial measurements of body weights in animals harboring Glio12421 tumors treated with vehicle and twice daily doses of BRD3800. C. Serial measurements of body weights in animals harboring Glio11305 tumors treated with vehicle and twice daily doses of BRD3800.
